# Twin-to-Twin Transfusion Syndrome: Diagnostic Imaging and Its Role in Staving Off Malpractice Charges and Litigation

**DOI:** 10.3390/diagnostics11030445

**Published:** 2021-03-04

**Authors:** Simona Zaami, Gabriele Masselli, Roberto Brunelli, Giulia Taschini, Stefano Caprasecca, Enrico Marinelli

**Affiliations:** 1 Department of Anatomical, Histological, Forensic, and Orthopedic Sciences, Sapienza University of Rome, 00161 Rome, Italy; enrico.marinelli@uniroma1.it; 2 Radiology Department, Umberto I Hospital Sapienza University, 00161 Rome, Italy; gabriele.masselli@uniroma1.it; 3 Department of Maternal and Child Health and Urological Sciences, Sapienza University of Rome, 00161 Rome, Italy; roberto.brunelli@uniroma1.it; 4 Diagnostic Radiographer, Radiology Department, Casa di Cura Pio XI, 00165 Rome, Italy; giulia.taschini@gmail.com; 5 UOSD Department of Emergency Radiology—Azienda Policlinico Umberto I, “Sapienza” University of Rome, 00161 Rome, Italy; stefano.caprasecca@uniroma1.it

**Keywords:** twin-to-twin transfusion syndrome (TTTS), diagnostic imaging, malpractice, litigation

## Abstract

The study aims to expound upon the imaging-based diagnostic methodologies aimed at identifying twin-to-twin transfusion syndrome (TTTS), a serious, somewhat rare prenatal condition that takes place in pregnancies where identical twins, or other multiples, share a placenta (monochorionic placenta), highlighting how medico-legal outcomes can be affected by provable compliance with consolidated diagnostic guidelines or best practices. It is of utmost importance to produce a prompt identification of TTTS instances; an early diagnosis is in fact critical in order to effectively treat and manage TTTS. By virtue of TTTS being a highly progressive condition, a delay in diagnosis can result in disastrous outcomes; just a few weeks delay in the diagnosis of TTTS can turn out fatal for one or both twins. Hence, most TTTS malpractice claims involve allegations of medical negligence, namely the failure to recognize the condition in a timely fashion, or to proceed with adequate diagnostic and therapeutic pathways. In that regard, case law databases have been pored over (Justia, Lexis, Leagle), and five significant court cases have been examined and discussed in an attempt to identify objective medico-legal standards and bring to the forefront relevant forensic dynamics. In fact, when health professionals are capable of proving adherence to guidelines or best practices, this can shield them from malpractice allegations and ensuing litigation.

## 1. Introduction

Twin-to-twin transfusion syndrome (TTTS) results from a hemodynamical imbalance of placental vascular anastomosis connecting the circulation of the two fetuses, whose consequence is the presence of hypovolemia, oliguria, and oligohydramnios in a twin (donor) and hypervolemia, polyuria, and polydramnios in the co-twin (recipient). The condition typically develops during the 15–26 week gestation period. Abnormalities in blood vessel connections can form in the placenta, thus causing blood to flow between the fetuses in an uneven fashion. The “donor” twin therefore becomes dehydrated, whereas the recipient twin develops high blood pressure and produces too much urine, leading to the overfilling of the amniotic sac. Moreover, the donor twin is often smaller, with a birth weight 20% less than the recipient′s birth weight. Mortality from severe untreated TTTS is extremely high, with reported rates ranging from 70% and 100% [1]. Deaths occurring in utero are usually to be ascribed to fetal cardiac failure. Without treatment, pre-viable or extremely preterm births contribute to high perinatal mortality. Of twins that are liveborn, a significant proportion suffer from postnatal complications of TTTS, including heart and kidney dysfunction and complications of polycythaemia and anaemia, or neurological and cardiological consequences [2].

TTTS usually occurs in the second trimester and is a dynamic condition that can remain stable, occasionally regress spontaneously, or evolve quickly. It affects 10–15% of monochorionic–diamniotic (MCDA) twin gestations [3] and also affects a similar proportion of the MCDA twin pairs in dichorionic–triamniotic (DCTA) triplet gestations [4]. Other studies have found that monochorionic twin placentation occurs in 20% of spontaneous twin pregnancies and, interestingly, almost 5% of those are obtained by medically assisted reproduction [5,6,7].

Monochorionic twin pregnancy can be defined as a congenital anomaly of the placenta, due to which the twin vessel systems communicate through vascular anastomoses on the placental surface. It is the angioarchitecture of these vascular anastomoses that determines the risk profile [8]. Transfusion between twins is therefore a normal event; in fact, the bidirectional artery-artery or vein-vein anastomoses compensate for the hemodynamic imbalance caused by the smaller and deeper one-way arteriovenous anastomoses, but when there is unequal hemodynamic and amniotic fluid balance, it can cause the risk of developing TTTS [5].

## 2. Screening for TTTS

In monochorionic twin pregnancy, ultrasound screening for TTTS includes a checkup every two weeks starting during the second trimester of gestation until the end of pregnancy [9].

Ultrasound assessment should include fetal biometry, visualization of bladder filling, measurement of amniotic fluid, evaluation of Doppler flowmetry in the umbilical artery and venous duct, and evaluation of peak systolic velocity in the MCA in both twins [10].

The diagnosis of TTTS requires the presence of significant amniotic fluid imbalance. TTTS is define according to the sonographic criteria of oligouric oligohydramnios in the donor twin with deepest vertical pocket (DVP) of 2 cm or less and polyuric polyhydramnios in the recipient with DVP of 8 cm or greater prior to 20 weeks and 10 cm or greater after 20 weeks [7]

TTTS is associated with a high risk of brain injury in survivors, and the incidence and severity of injury is related to the Quintero stage [11,12], which includes a five-stage classification correlating the combined ultrasound assessment with the flowmetric data of the umbilical vein and venous duct Doppler study (Table 1). It is worth noting that, although the Quintero staging system provides a helpful definition of this progression, TTTS is not always linear and can develop rapidly [13,14].

There are some discrepancies concerning the validity of Quintero staging of TTTS. It has been noted that Stage-I disease is not necessarily associated with the best outcomes. For example, some recipient twins in pregnancies categorized as Quintero stage I of TTTS may have a degree of cardiac dysfunction. Another criticism is that it does not represent a chronological order of deterioration; e.g., Stage 1 can become Stage 5 without passing through Stages II, III, and IV, and it does not predict survival well after treatment [11]. Nevertheless, the Quintero staging system remains the one most widely used for the classification of twin pregnancy complicated by TTTS.

## 3. Magnetic Resonance Imaging

Although ultrasound (US) is the method of imaging of I and II instance in the fetal-placental evaluation, attesting both as a screening investigation and as a II level method if an anomaly is found, magnetic resonance imaging (MRI) can greatly improve the diagnostic performance of US during pregnancy, by allowing for a further visualization of the alterations affecting the fetuses, due to its capabilities and the non-use of ionizing radiation [15]. The numerous hardware and software developments to which MRI has been subjected over the years, aimed at obtaining a greater acquisition speed, limiting fetal movement, and increasingly optimizing tissue characterization, have made it a diagnostic method suitable for imaging feto-placental and all the appendages that support its growth, including the placenta, proving it to be an alternative imaging modality that could offset the limits of ultrasound [16,17]. Fetal MRI is currently performed in most centers with a 1.5 T field. An adequate signal-to-noise ratio will be obtained at this field strength. To date, non-deleterious effects at short-term and long-term on developing fetus have been proven with exposure fields equal to or less than 1.5 T. The greatest theoretical risk on the developing fetus would be during organogenesis in the first trimester. Safety issues include the possible biological effects of the static magnetic field and the risks associated with gradients, radiofrequencies, and exposure times. Similarly, the use of contrast media, gadolinium agents, are generally not recommended in pregnancy at any stage [18]. The reason for this lies in its long half-life, with repeated cycles of excretion and reabsorption by the fetus; in fact, gadolinium crosses the placenta and is excreted from the urinary tract of the fetus in the amniotic cavity and subsequently reabsorbed, thus prolonging the elimination time. Furthermore, the gadolinium-chelate molecules remain in the amniotic fluid for an undetermined amount of time, during which they can undergo dissociation, releasing free toxic ions in the amniotic fluid [19].

**Figure 1:**
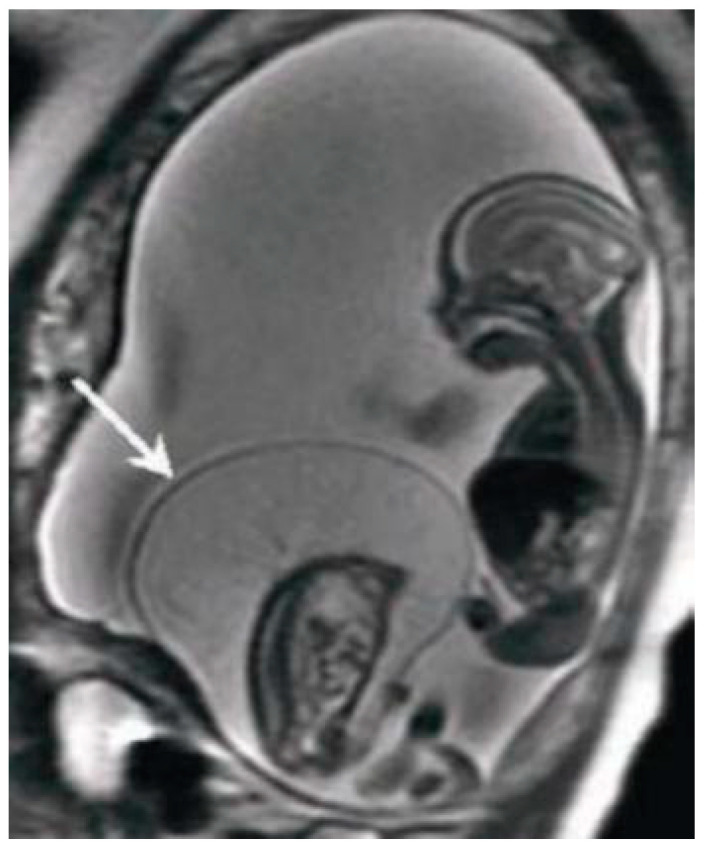
Fetal magnetic resonance imaging (MRI) of Twin reversed arterial perfusion (TRAP) sequence. Half-Fourier Acquisition Single-Shot Turbo-Spin-Echo (HASTE) T2 Sag. Both the acardiac (signaled by the arrow) and pump twins are visible.

MRI has the greatest advantage in the second and third trimesters of pregnancy to minimize the difficulties due to small dimensions and frequent fetal movements in early management periods, particularly in the third trimester, and it is therefore optimal for the evaluation of anomalies in charge of the cerebral cortex, thanks to a better spatial resolution. Fetal MRI prior to 20 weeks is uncommon. Feto-placental imaging involves the acquisition of different sequences performed on the three planes of space, axial, coronal, and sagittal, some of which are indispensable and others are optionally added depending on the clinical question. The development of fast sequences has allowed for the reduction of motion artifacts, with the possibility of acquiring a series of images in 15/20 s and having a complete acquisition of the exam in around 20–40 min. The MRI examination is preceded by the acquisition of a localizer on the three planes; it is an extremely rapid sequence, of non-diagnostic quality, which allows verification of the position of the placenta in craniocaudal and lateral-lateral and to evaluate the correct coil positioning and identification of landmarks for subsequent acquisitions. Generally, the field of view (FOV) used varies within the 30–40 cm range, and the slice thickness is around 4–6 mm; thin slices of 2–3 mm can be obtained, but the smaller the slice thickness, the lower the signal-to-noise ratio (SNR). Overall, the choice of slice thickness always depends on the management age and therefore on the size of the fetus. The typical matrix is 256 × 256 [12]. The sequences used for feto-placental imaging are the T1 and T2 dependent sequences acquired during respiratory apnea. The T2-weighted sequences provide a greater contrast resolution between the various structures present within the field of view, between the placenta, the amniotic fluid, and the fetuses, which, being structures with different tissue compositions, show a different signal intensity in response to the radio frequency (RF) stimulus (Figure 1).

The dependent T1 sequences, sent together with fat suppression, are used for the evaluation of blood collections, index of placental hematomas, or placental detachments [20]. Among the sequences added according to the clinical question, diffusion weighted imaging (DWI) MRI with weighted sequence in diffusion provides information at a microscopic level, enabling clinicians to explore the tissue microarchitecture in a non-invasive way. The contrast of the image depends on the movement, microscopic and random, of water protons that can be altered in different pathological processes [21,22].

### Diffusion Weighted Imaging

**Figure 2:**
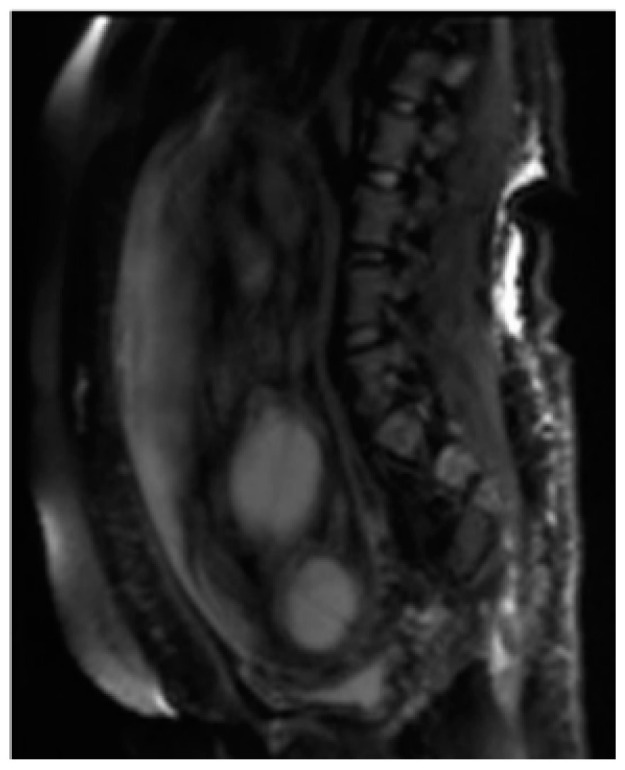
Diffusion Weighted Imaging (DWI).

Among the sequences added according to the clinical question, diffusion weighted imaging (DWI) MRI with weighted sequence in diffusion provides functional information and qualitative and quantitative analysis at the microscopic level of the diffusion of water molecules within the placenta, allowing the clinician to explore the tissue microarchitecture in a non-invasive way (Figure 2).

The contrast of the image depends on the microscopic and random movement of the water protons, which can be altered in different pathological processes [23]. The diffusion sequence is generally performed last as it involves the application of powerful gradients that cause a dephasing and a subsequent rapid rephasing of the spins, causing a stimulation in the fetuses that are often agitated [24].

TTTS typically affects the health of identical twins dramatically. Prematurity and neurodevelopmental impairment affect both the donor and recipient twins; cardiovascular failure and obstruction of the right ventricular outflow tract are typical complications of recipients, possibly resulting in long-term morbidity [25]. Some of the common complications associated with Twin-Twin Transfusion Syndrome include:

- Donor twin anemia;
- Recipient twin heart failure caused by the heart pumping excessive levels of blood;
- Recipient twin brain defects, heart problems, and digestive or respiratory health issues;
- Preterm labor caused by pregnancy induction or ruptured membranes;
- Donor twin compromised fetal development;
- Recipient twin health issues caused by excessive amniotic fluid that leads to premature membrane rupture;
- Heart damage (recipient twins may develop progressive biventricular hypertrophy and diastolic dysfunction, in addition to poor right ventricular systolic function, possibly leading to functional right ventricular outflow tract obstruction and pulmonic stenosis);
- Brain damage;
- Fetal death.

Moreover, most pregnancies involving TTTS never go to full-term. Because of that, many of these premature births share the same complications and long-term effects as other premature babies, which include [26,27]:

- Stunted physical development;
- Learning disabilities;
- Compromised communication skills;
- Asthma and other respiratory problems;
- Bronchopulmonary dysplasia (BPD), a chronic lung disease caused by abnormal growth or inflammation;
- Hearing loss;
- Vision problems, including retinopathy;
- Dental issues caused by delayed tooth growth that may result in crooked teeth or tooth discoloration;
- Increased risk of developing attention deficit-hyperactivity disorder (ADHD);
- Increased potential risk of sudden infant death syndrome (SIDS);
- Increased risk of developing chronic diseases including diabetes, hypertension, or heart disease.

Because of the increased risk of preterm birth, administering steroids for fetal maturation ought to be weighed at 24 to 33 6/7 weeks, particularly in pregnancies complicated by stage III TTTS and those undergoing invasive interventions. Two treatment options are currently available for TTTS treatment: amniocentesis aimed at draining excess amniotic fluid from the recipient twin, which reduces amniotic fluid levels in order to help improve blood circulation, and laser surgery, through the insertion of a thin, fiber-optic scope through the mother′s abdominal wall, through the wall of the uterus, and into the amniotic cavity of the recipient twin [28]. The blood vessels on the placental surface are thus examined directly with the scope, and the abnormal vascular connections between the twins can be spotted and eliminated through a laser beam. Only those vessels that go from one twin to the other are coagulated by the laser beam. The normal blood vessels that help nourish each twin are left intact. Laser photocoagulation of placental anastomoses is the recognized treatment of choice for Stages II–IV TTTS [29]. Upon completion of the laser procedure, an amnioreduction (i.e., removal of excess amniotic fluid) is carried out in order to lower the risk of early labor and help make the pregnancy less demanding. Survival of at least one fetus following laser surgery is currently > 90%; the increased use of interventions such as laser coagulation of the intertwin anastomoses (laser therapy) has resulted in significantly improved survival rates for both twins (69.5%) as well [30]. Nonetheless, the neurodevelopmental outcomes for survivors are as yet not fully understood. Prior to laser therapy, at least one in five survivors of TTTS had serious adverse neurodevelopmental outcomes (usually cerebral palsy). Neurological impairment among survivors following laser surgery is currently estimated to range from 4 to 31%, although long-term follow-up data are still limited [31]. Scientific evidence exists proving the superiority of fetoscopic laser ablation over serial amnioreductions regarding survival and neurological outcome for stages II–IV TTTS [32]. However, the optimal management of stage I is still undetermined and currently debated. Survival rates after fetoscopic laser surgery have sharply risen over the past 25 years. High volume centers report up to 70% double survival and at least one survivor in >90%. Long-term neurodevelopmental impairment occurs in about 10% of children after laser surgery [33]. As mentioned before, roughly ten percent of identical twins (and other identical multiple babies) develop TTTS. Typically, at least one monozygotic twin is affected when the fetus disproportionately shares a monochorionic placenta. In many cases, restricted nutrients to one twin might result in abnormal growth or death.

Any failure or delay to diagnose TTTS or treat the syndrome properly may result in medical malpractice lawsuits being filed [34]. Research has shown a disconnect between patients’ reporting TTTS symptoms and health-care providers responding accordingly and consistently, in the patient’ perception. It would be advisable for health-care providers to inform women pregnant with a monochorionic–diamniotic pregnancy to immediately report the presence of any symptom described in the present research, which may be associated with any number of complications associated with twin pregnancy [35]. Although serial ultrasonography is the only definitive way to diagnose TTTS, substantial efforts have been made to assess the value of maternal symptomatology reporting practices. Common maternal symptoms include a rapid and marked increase in a mother’s abdominal girth, due to the recipient’s expanded amniotic fluid compartment; an overly large uterus for the period; pain, tightness, or contractions in the abdomen; abrupt body weight gain; and bloating in the hands and legs in early pregnancy. Given the high-risk nature of monochorionic–diamniotic twin pregnancies, the full range of maternal symptoms during TTTS should be explored, and never discounted, in order to assist with differential diagnosis. Doctors and other health-care providers should inform women pregnant with monochorionic–diamniotic twin pregnancies to be alert for the presence of any symptom commonly associated with TTTS and immediately report such symptoms to their doctors. In addition, health-care providers must promptly look into any concerns and physical complaints and discomfort experienced by women during a monochorionic–diamniotic twin pregnancy. Delivering proper care and carrying out therapetic measures in a timely fashion may be essential; statistics for intrauterine laser ablation of placental vessels as treatment for TTTS show that there is a 70 percent chance that both twins will survive if treatment is prompt, although that rate is affected by the gestational age at procedure and the presence of abnormal Doppler findings [36].

## 4. Litigation Stemming from Negligence

Doctors may be held financially liable under tort law statutes if their actions were negligent and they failed to accurately diagnose fetal conditions. Doctors, in fact, might not have carried out the proper tests, identified symptoms, or followed up on early indicators that something was wrong, including:

There was an identifiable difference between each fetus′ umbilical cord size;

There were obvious differences in amniotic fluid and amniotic sac size;

The size of each baby was significantly different.

The provable failure to either test for or properly follow up on and treat the above-mentioned signs may indicate negligence. Case law analysis revolving around five instances of relevant TTTS litigation are laid out in Table 2.

## 5. Discussion

As many instances of TTTS-related lawsuits show, medical malpractice may contribute to injuries sustained by a child as a result of the negligent handling or treatment of TTTS or a failure to timely diagnose the condition. Obstetricians, gynecologists, or other physicians in charge of choosing and providing the most suitable forms of treatment for pregnant women are held to a high standard of care when it comes to the health and safety of mothers and fetuses alike. When a woman is pregnant with more than one identical fetus, her treating physician should be vigilant in performing certain tests aimed at determining whether the pregnancy may be complicated by TTTS. Any failure on the part of doctors to conduct these tests may form the basis of a medical malpractice claim if the tests could have prevented TTTS-related injuries. Medical malpractice law applies when a medical professional fails to use the level of care that a reasonably prudent medical professional would have used in the same or similar circumstances, thereby causing injuries or even death to the patient. It is important to note that the onus is on the plaintiff to establish malpractice in any case, and each of the following elements must be determined by a preponderance of the evidence; however, particularly under tort law statutes, compliance with cautionary measures must be proven by doctors. Such patterns may be summed up as: Provided that the defendant medical professional owed the patient a duty of care, then the defendant doctor failed to live up to the duty of care owed to the patient, and the defendant medical professional’s breach was a direct and proximate cause of the patient’s injury; i.e., a causal relationship can be proven.

In addition to stressing the essential nature of a properly developed informed consent process and individually tailored patient assessment of risk factors, it cannot be overlooked that a court of law, particularly in tort law, will likely tend to hold professionals and facilities liable (thus concluding that malpractice stemming from negligence has occurred, rather than mere unavoidable complications) if any inconsistencies are found in the informed consent documentation and patient records. Furthermore, the role of clinical practice guidelines is twofold when it comes to medical malpractice claims. They can in fact be brought up as a defensive value; i.e., exculpatory evidence in legal proceedings by a health professional or as inculpatory evidence by plaintiff patients damaged by an alleged standard of care breach [41]. Determining whether such a breach actually occurred is as essential as it is complicated [42] in medical malpractice claims.

## 6. Conclusions

Overall, any failure to adhere to surgical safety protocols, clinical practice guidelines, and best practices, and to properly produce documentation reflecting such adherence, will most likely result in courts ruling against healthcare providers and facilities. To that end, it is of utmost importance to devise sets of standardized, clearly defined guidelines and best practices, both for the sake of patients and to shield healthcare professionals from adverse court decisions. There is no denying, in fact, that medical malpractice entails substantially higher direct, as well as indirect, health care costs, such as high insurance premiums, hefty expenses for compensatory damages, litigation fees, and “defensive medicine” practices. Virtually all of the TTTS-related litigation herein outlined has stemmed from a failure to put in place diagnostic measures in a timely fashion. Lastly, hospital protocols and policies are themselves key elements in keeping professionals and facilities from being subjected to malpractice allegations and lawsuits. It is in fact worth noting that, in some instances, a hospital or other employer may also be liable for a medical professional’s negligence and ensuing malpractice-related damages sustained by patients under the legal concept of “vicarious liability”. Liability also may be established when the hospital is found to have been negligent in adopting policies and procedures for the care and treatment of TTTS patients or mismanaged the process of hiring, training, screening, monitoring, or retaining a negligent professional.

## Figures and Tables

**Table 1:** Quintero stage system.

Stage	Classificationfication
I	In the early stage of twin-to-twin transfusion syndrome (TTTS), there is no amniotic fluid or reduced volumes surrounding the donor twin and increased fluid surrounding the recipient twin. Polyhydramnios–oligohydramnios sequence: Deepest vertical pocket (DVP) > 8 cm in recipient twin and DVP < 2 cm in donor twin.
II	The donor twin can be viewed with an empty bladder and possibly the bladder is not visible on the ultrasound.
III	Absent or reversed umbilical artery diastolic flow: abnormal blood flow levels are provided by the umbilical cord to one or both twins. The stage also confirms fetal ductus venosus, by which the flowing blood in a portion of the left umbilical vein is shunted to the inferior vena cava (IVC).
IV	Hydrops in one or both twins (i.e., abnormal fluid accumulation in more than one body cavity). Additionally, swelling is at times identified in the skin surrounding the head and around the heart, the lungs, and in the abdomen.
V	Death of one or both twins. A confirmed diagnosis of an increased progression of the syndrome often results in the donor twin′s poor prognosis of survival.

**Table 2:** Relevant case law involving TTTS (Sources: Justia, Lexis, Leagle, spanning 2000–2020).

Location/Date	Case Specifics	Legal Outcome
Fulton County, GA, USA, 2003	A patient expecting twins, with a history of preterm labor, was admitted to a hospital at 31 weeks’ gestation. A biophysical profile showed a score of 8/8 for one twin and 6/8 for the other, with absent end-diastolic flow. The doctor ordered a repeat biophysical study for the following day. Prior to the test, however, one fetus developed a terminal bradycardia. Emergency delivery was performed, but the newborn died as a result of TTTS. The other twin spent several weeks in the neonatal intensive care unit and survived with no complications. The mother contended that the doctor should have repeated the biophysical test sooner or placed the twins on electronic fetal monitoring, and that better monitoring would have alerted the doctor to the twin’s failing condition, prompting delivery in time to save him, which the doctor denied.	The jury returned a defense verdict, by which no recovery was awarded [37].
Delaware County, PA, USA, 2007	The plaintiffs alleged that severe neurological injuries to one newborn and the death of the other were caused by the failure of the defendant physicians to diagnose and treat twin-to-twin transfusion syndrome (TTTS). Blood was transfused from the “donor” twin to its “recipient” sibling such that the donor became growth restricted while the recipient developed circulatory overload with associated complications. Plaintiff’s mother was 10 weeks pregnant when an ultrasound revealed a twin gestation. Unfortunately, the radiologist interpreting that ultrasound failed to determine whether the twins shared a placenta. A second ultrasound, performed eight weeks later, noted an 18% discrepancy in the weight of the twins, a large discrepancy in their abdominal circumferences, and an amniotic fluid imbalance, all signs and symptoms of TTTS. Unfortunately, the defendant obstetricians failed to take action to monitor and treat the evolving syndrome.	The case was strongly contested by the defendants, who summoned expert witnesses from the fields of obstetrics, radiology, pathology, pediatric neurology, perinatology, pediatric neuropsychology, and forensic economics to testify not only that there were other possible causes of injury to the minor-plaintiff, but also that treatment options for TTTS, if present, were experimental in nature and would not likely have avoided injury to the plaintiff. The settlement will be held in trust for the 5-year-old plaintiff to meet her future needs. After selecting a jury, attorneys negotiated a $2.3 million settlement [38].
Pardini v Allegheny General, Pennsylvania, USA, 2007	A father filing a separate claim for emotional distress arising from the death of his twins. He alleged that the defendant hospital and doctors failed to properly diagnose and manage twin transfusion syndrome, which ultimately led to the stillbirths.	The case settled for $125 k. The mother had already filed a claim on behalf of the deceased twins and was awarded a separate undisclosed settlement [39].
James Cook University Hospital, Middlesbrough, UK, July 2015	In July, Ms Jaffray began experiencing severe pain in her ribs and back and attended the James Cook University Hospital, Middlesbrough. She was diagnosed with a pulled muscle, but when the pain became more severe, the couple re-attended the hospital’s maternity unit and got the same diagnosis. She was sent home without a scan. The following day, she was hospitalized again with severe pain. An ultrasound scan was performed that day, showing that her unborn babies were suffering from TTTS. Ms Jaffray was given two options, the first being a Caesarean section providing virtually no chance of survival for the twins, or secondly, to undergo ‘intrauterine laser ablation of placental vessels.’ Ms Jaffray opted for the latter. Unfortunately, in the UK, there are relatively few specialists in this kind of invasive fetal surgery. As a result, Ms Jaffray had to be transferred to St Georges Hospital, London. One of the babies died that night and the other died the following day while Ms Jaffray was being transferred for specialist treatment.	The South Tees NHS Hospitals Trust has apologized to the couple and conceded that if Ms Jaffray had undergone an ultrasound scan when she first presented with abdominal pain on 22 July 2015, signs of TTTS could have been detected and transfer to the specialist center for laser treatment could have been arranged sooner. No matter how serious the case and although the South Tees NHS Foundation Trust has admitted and apologized for its failings, in order to succeed in a clinical negligence case, it would have to be shown, by way of expert evidence, that had the scan been performed and the diagnosis been made on Ms Jaffray’s first presentation on 22 July 2015, there would have been a 51 percent or greater chance that the babies would have survived. The case was settled for an undisclosed amount [40].
J.A.S. v Cambridge Pediatrics (filed in Pennsylvania, USA, in 2016	A hospital and pediatrician were sued for the negligent mismanagement of a pregnancy and the premature delivery of identical twins suffering from TTTS. One of the twins was stillborn and the surviving twin was extremely premature with a birth weight of only 2 lbs (0.907 kg) and an Apgar score of 5. Hours after birth the surviving newborn suffered severe intracranial hemorrhage, catastrophic brain damage, and ensuing severe disability.	The defendants admitted negligence and the case went to trial solely on the issue of damages. The jury awarded compensatory damages of $8.4 mil [33].
